# At the interface of community and healthcare systems: a longitudinal cohort study on evolving health and the impact of primary healthcare from the patient's perspectiv

**DOI:** 10.1186/1472-6963-10-258

**Published:** 2010-09-03

**Authors:** Jeannie Haggerty, Martin Fortin, Marie-Dominique Beaulieu, Catherine Hudon, Christine Loignon, Michel Préville, Danièle Roberge

**Affiliations:** 1 Faculty of medicine, McGill University, Montreal, Canada; 2 Department of Family Medicine, Université de Sherbrooke, Sherbrooke, Canada; 3 Centre de recherche du Centre hospitalier de l'Université de Montréal, Montréal, Canada; 4 Faculty of Medicine, Université de Sherbrooke, Sherbrooke, Canada; 5 Centre de recherche de l'Hôpital Charles LeMoyne, Longueuil, Canada

## Abstract

**Background:**

Massive efforts in Canada have been made to renew primary healthcare. However, although early evaluations of initiatives and research on certain aspects of the reform are promising, none have examined the link between patient assessments of care and health outcomes or the impacts at a population level. The goal of this project is to examine the effect of patient-centred and effective primary healthcare on the evolution of chronic illness burden and health functioning in a population, and in particularly vulnerable groups: the multi-morbid and the poor.

**Methods/Design:**

A randomly selected cohort of 2000 adults aged 25 to 75 years will be recruited within the geographic boundaries of four local healthcare networks in Quebec. At recruitment, cohort members will report on socio-demographic information, functional health and healthcare use. Two weeks, 12 months and 24 months after recruitment, cohort participants will complete a self-administered questionnaire on current health and health behaviours in order to evaluate primary healthcare received in the previous year.

The dependent variables are calculated as change over time of functional health status, chronic illness burden, and health behaviours. Dimensions of patient-centred care and clinical processes are measured using sub-scales of validated instruments. We will use Poisson regression modelling to estimate the incidence rate of chronic illness burden scores and structural equation modelling to explore relationships between variables and to examine the impact of dimensions of patient-centred care and effective primary healthcare.

**Discussion:**

Results will provide valuable information for primary healthcare clinicians on the course of chronic illness over time and the impact on health outcomes of accessible, patient-centred and effective care. A demonstration of impact will contribute to the promotion of continuous quality improvement activities at a clinical level. While considerable advances have been made in the management of specific chronic illnesses, this will make a unique contribution to effective care for persons with multiple morbidities. Furthermore, the cohort and data architecture will serve as a research platform for future projects.

## Background

Canadian provincial and federal health commissions have concluded that a strong primary healthcare foundation is the key to a sustainable health system [1-6]. Ecologic studies suggest that regions with robust support in primary healthcare have better health indicators, such as longer life expectancy, lower all-cause mortality, better health equity [7-13] and show better intermediate outcomes of care.

For the purposes of this research we use "primary healthcare" in its narrow sense to refer to primary medical care, provided in organizational models composed minimally of family physicians or generalists, who may or may not be working with other health and social services professionals.

The goal of primary healthcare is to optimize health and functional health through activities of timely diagnosis and treatment, clinical disease prevention, health promotion and support during rehabilitation and palliative care. Through effective care, clinicians can help patients adopt more positive health behaviours [14,15], avoid morbidity [16-18] and improve functional health even in the face of prevailing disease [15]. As patient-centeredness is the core value of care delivery among primary healthcare professionals [19,20], this research includes dimensions of primary healthcare identified as being "person-oriented": [21] accumulated knowledge of the person (relational continuity), interpersonal communication, shared decision-making, and respectfulness.

While quality and effectiveness of healthcare is usually measured by provider's compliance with established norms for defined conditions, this research focuses on quality as it relates to the person-centeredness of clinical processes and approaches and their impact on functional health and health behaviour [22-25].

Functional health is the extent to which an individual perceives that physical or mental health limits his/her capacity to carry out daily activities and social roles [26,27]. Functional health declines with increasing chronic illness burden [28-30]. The repeated Canadian Community Health Surveys (CCHS) demonstrate that the population prevalence of chronic illnesses is increasing [31,32] but provide little insight about how the increase of illness burden in individuals is dynamically related to functional health. Functional health can improve through self-management and self-efficacy to change negative health behaviours and adopt positive ones [30,33].

Although health behaviour change is influenced by multiple factors, there is relatively strong evidence that physicians' recommendations and reinforcement have a strong influence on such changes, e.g. smoking [34,35], alcohol consumption [36-38], regular exercise [39-42] and healthy weight. In this research we will study both health behaviour status and intention to change.

There are groups of patients who are at high risk of health deterioration and may be particularly vulnerable to problems in the organization of healthcare such as the multi-morbid and the poor and consequently, most likely to benefit from patient-centred care. Our rough estimates suggest that the population prevalence of multi-morbidity increases by 1% per year of age [43-47]. Multi-morbidity is a major issue for primary healthcare providers [43] as research shows clear associations between multi-morbidity and the experience of unfavourable outcomes. We also found a clear association between illness burden and functional health and psychological distress [48-52].

Poverty is a state of material and/or social deprivation that limits the capacity to mobilize resources to achieve well-being [53,54]. The way care is delivered has a large impact on the effectiveness of care that the poor receive. Although ecologic studies suggest that primary healthcare can improve health inequity in the population [7,9-13], the demonstration for what impact this may have can only be made through the longitudinal follow-up of individuals.

The underlying premise of this research is that patient-centred and effective primary healthcare can maximize functional health, in general, and particularly in vulnerable groups, such as those with a high burden of chronic illnesses and the poor. We will longitudinally follow the health and healthcare experience of individuals: 1) to describe changes in functional health, chronic illness burden and health behaviours; 2) to examine the impact of patient-centred and effective primary healthcare on functional health and other outcomes of interest (health behaviours, chronic illness burden, health service utilization); and, 3) to explore the relationships between intermediate outcomes and individual characteristics, and functional health.

## Methods/Design

The proposed study is a cohort of 2000 adults aged 25-to-75 years followed for 4 years. The target population is community-dwelling adults undifferentiated by disease, who would seek primary healthcare locally, do not suffer from major cognitive impairment, and are able to respond to written and oral questions in English or French. Participants will be randomly selected within the geographic boundaries of four local healthcare networks in metropolitan, rural and remote urban agglomerations of Québec. At recruitment (T_0_), cohort participants will report on socio-demographic information, functional health and healthcare use. Two weeks (T_1_), 12 months (T_2_) and 24 months (T_3_) after recruitment, they will complete a self-administered questionnaire on their current health, health behaviours and primary healthcare experience in the previous year. Use of medical services will be confirmed through the review of administrative databases.

Participants will be recruited through a telephone survey with a two-stage sampling design. Following first contact, staff will select the adult in the household with the most recent birthday [55]. Participant contact information will be sent to the research team (independently of data), who will then mail a "welcome package" containing a consent form, questionnaires and a postage-paid return envelope. Phone contact will follow to review the consent form and respond to questions.

### Follow-up and cohort maintenance

The principal threat to the internal validity of a cohort design is the differential loss to follow-up. We will optimize cohort maintenance and subject retention by using newsletters and greeting cards but do expect some attrition between recruitment and the return of the T_1 _questionnaire, and over time [55]. To have 2000 subjects at T_3, _we will initially over-recruit by 20% (2400 at T_1_).

### Data collection

#### Patient self-report questionnaires

##### T_0 _

Demographic data and information on functional health and use of health services over the previous year collected at T_0 _will reduce the later response burden and provide valuable information on patients lost to follow-up, namely age, gender, language, education, perceived income adequacy, usual source of primary healthcare and the strength of affiliation, and overall assessment of health.

##### T_1_, T_2_, and T_3_

The self administered questionnaire containing approximately 160 questions will be available on paper (mailed) or internet. Since online responding allows for immediate data capture and built-in quality checks, we will strongly encourage this modality [56]. Respondents with chronic diseases will respond to an additional set of 32 questions. Overall, it takes approximately 50-70 minutes to complete (general vs. chronic disease) [57]. We used validated subscales where possible, as outlined in Table 1, and described in detail below for key components.

**Table 1:** Operational definitions, measurement instrument, and available metric properties of primary and secondary outcomes of interest

Variable	Measurement Instrument
**Functional Health**	Elicited in T_0 _questionnaire using the SF-12v2 (13 items). The questions generate a physical and mental component, each with a theoretical range of 0 to 100. A difference of 5 points between persons or over time is considered clinically significant. The instrument has solid psychometric properties and was validated for French Canadian subjects [60,61].

**Number and Severity of Chronic Conditions** 1. Hypertension 2. Elevated cholesterol 3. Asthma 4. Pulmonary problem 5. Diabetes 6. Thyroid disorder 7. Osteoarthritis 8. Rheumatoid arthritis 9. Back pain or sciatic pain 10. Osteoporosis 11. Other illness that affects the members or the articulations 12. Reflux, peptic ulcer or pyrosis 13. Intestine problem 14. Overweight 15. Hearing problem 16. Vision problem 17. Cardiac illnesses 18. CVA 19. Heart failure 20. Cancer (past 5 years) 21. Depression or anxiety problems 22. Other chronic health problems not mentioned above	Disease Burden Morbidity Assessment [64] (22 items). Subject is asked to report whether a health professional has diagnosed the listed condition and to estimate the extent to which it interferes with his daily activities on a Likert scale ranging from "not at all" (1) to "a lot" (5). 0 = condition not diagnosed. Though theoretically scores range from 0 to 125, in practice the maximum is 50 [64]. Compared to chart reviews, self reports of conditions have a median sensitivity of 75% and specificity of 92%. Self-reported scores correlate more closely to functional health than chart measures of multi-morbidity [64]. No population norm exists but in elderly persons the average score was 13.9 with a maximum of 51 [64]. Multi-morbid individuals are those with a score of ≥10. Compared to chart reviews, self reports of conditions have a median sensitivity of 75% and specificity of 92%. The score correlates more closely to functional health than chart measures of multi-morbidity [64].

**Health Behaviours**	

Body Mass Index: BMI	Height, weight (2 items). Normal Body Mass Index (BMI) = 19-24.9 kg/m². Scoring ranges from 1 (ideal weight for height) to -3 (morbidly obese BMI > 35 kg/m²). Among those above ideal weight: intention to engage in weight change over the next 6 months. [68,72] (1 item).

Fruit and vegetable consumption	*Enquête Saguenay-Lac-St-Jean 2007 *[66]and other regional surveys (3 items). Intention to consume at least 3 daily servings of fruit and vegetables over the next 6 months [68,72] (1 item).

Smoking: status, age-onset of daily smoking, current intensity, nicotine dependency	CCHS 3.1, Daily smoking, daily number (same as CCHS p. 110-116). Found to be highly reliable and valid [84] (2 items). Scoring ranges from 1 (< 100 cigarettes in lifetime) to -3 (daily smoking ≥ 20 cigarettes). *Enquête Saguenay-Lac-St-Jean 2007 *[66]and other regional surveys: ever smoke (1 item). Among smokers: intention to quit smoking over the next 6 months [68,72] (1 item).
Quit history, attempts and intentions	CCHS p.117 (2 items).
Receipt of smoking cessation advice and aid from health professional	CCHS p. 120 (3 items).
Second-hand smoke exposure	CCHS p.124 (2 items).

Physical activity	*Enquête Saguenay-Lac-St-Jean 2007 *[66]and other regional surveys (4 items): Practice of regular physical activity. Intention to engage in regular active exercise (at least 3 times per week for 20 minutes per time) over the next 6 months [68,72] (1 item).

Alcohol use-frequency, problem drinking	*Enquête Saguenay-Lac-St-Jean 2007 *[66]and other regional surveys (4 items). Adapted from CCHS 3.1 (driving under the influence) (1 item). Among problem drinkers: intention to reduce alcohol consumption over the next 6 months [68,72] (1 item).

**Psychological distress: **the general concept of maladaptive psychological functioning in the face of stressful life events	K6 [85] -Frequency of feelings of tiredness, nervousness, hopelessness, restlessness, depression on a Likert scale from 1 (all of the time) to 5 (none of the time). CCHS p. 181 (6 items). Though not specific to any particular psychiatric disorder" most psychiatric patients score high on these measures and it discriminates well between mental illness severity [85,86] α = 0.89.

**Use of specialists and specialty testing for common conditions**	Administrative database.

**Hospital emergency room use**	Administrative database.

We will apply the Dillman method [58] to maximize response to questionnaires at T_1_, T_2 _and T_3_: a personalized reminder/thank you note (postal or e-mail) at 2 weeks, followed by a re-mailing of the questionnaire to non-responders at 4 weeks, followed by a reminder to continued non-responders at 6 weeks and a phone call at 8 weeks. Compensation will be mailed with the questionnaire to enhance response [59]. Subjects will be considered lost-to-follow-up after eight weeks of non-response or explicit refusal to continue to participate.

#### Administrative medical services

We will use administrative medical services data from the Quebec healthcare insurance agency (RAMQ) to identify emergency room visits, hospitalizations and specialist visits, as secondary outcomes of interest.

### Outcomes of interest

**Figure 1:**
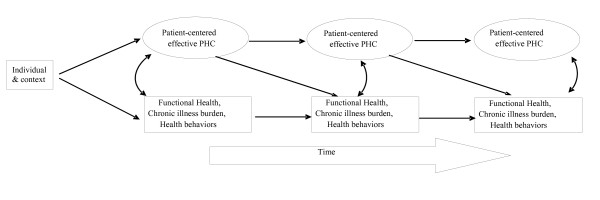
**Conceptual Model**.

The advantage of a prospective cohort design is the capacity to examine multiple outcomes of interest; some of which may be conceived as independent or mediating variables for other outcomes. Due to space limitations, we only provide operational definitions for functional health, our main dependent variable and two other intermediate outcomes: chronic illness burden and health behaviours. Figure 1 displays the conceptual model of the study. Table 1 provides an overview of the operational definition and available metrics of all outcomes measured when it applies.

#### Functional health

The main dependent variable in this project is functional health status measured with the second version of the Short-Form-12 survey (SF-12v2) [60,61]. It distinguishes between degrees of good health and poor health [62] and is sensitive to mild changes in illness burden [63]. It will allow us to examine the physical component, the mental component and overall assessment of health status separately. Functional health status is elicited by questions on physical health (physical functioning, role limited by physical capacity, bodily pain, overall health) and mental health (emotional health, vitality, social functioning, role limited by emotional state) in the last 4 weeks.

#### Chronic illness burden

We will measure illness burden using the validated Disease Burden Morbidity Assessment as this tool provides us with more sensitive and specific data than chart reviews [64]. For each of 22 physical and mental conditions diagnosed by a health professional, the person reports the extent to which the illness interferes with daily activities. Changes in score reflect both number of diseases and their perceived impact on daily living; consequently, both increases and decreases can occur over time.

#### Health behaviours

We will measure the presence and intensity of health behaviours (vegetable consumption, smoking, alcohol consumption, healthy weight and physical activity) using validated sub-scales from the Behaviour Risk Factor Surveillance System Questionnaire [65], from the *Enquête Saguenay-Lac-St-Jean 2007 *[66] and other regional surveys, and the CCHS questionnaire. We propose a summary score of health behaviour status, with negative scores for negative health behaviours and positive scores for positive health behaviours, ranging from -8 to 6. In addition, we will measure self-reported intent to engage in or adopt each healthy behaviour, using a single-item five-point response scale [67,68] that maps validly to the stage-of-change model [69] and has been linked to both functional health and future behaviour [70-72]. The intention scores used in this model predict long-term behaviours and are less labile than actual behaviours.

### Predictor variables

The main independent variable of interest is the patient's reported experience on the different dimensions of patient-centred and effective primary healthcare received from the regular provider over the previous 12 months, elicited at T_1_, T_2_, and T_3_. In addition, we are interested in the confounding and modifying effects of individual characteristics, especially multi-morbidity and poverty, but also other characteristics such as age effects and social support. The operational definition, sub-scale and available metric properties of these variables are outlined in Table 2.

**Table 2:** Operational definitions, measurement instrument, and available metric properties of predictor variables

Variable	Measurement Instrument
**Patient-centred care**	The score for each care dimension is calculated as the mean and then standardized to a 0-to-10 metric.

Relational continuity: A therapeutic relationship between a patient and one or more providers that spans various health care events and results in accumulated knowledge of the patient and care consistent with the patient's needs.	Duration of the relationship - <1 year, 1-3 years, > 3 years (1 item). Primary Care Assessment Survey (PCAS)[74]. Contextual Knowledge of Patient sub-scale (5 items). Rating of regular doctor's knowledge of whole medical history, personal situation, and values on a Likert scale from "poor" (1) to "excellent" (6). α = 0.90.

Interpersonal Communication: The ability of the provider to elicit and understand patient concerns, explain healthcare issues.	PCAS Communication Scale - (6 items). Rating of quality of thoroughness of history taking, listening skills, explanations on a Likert scale from "poor" (1) to "excellent" (6) α = 0.93.

Shared-decision making and empowerment: Ongoing cooperative process between patients and providers to define goals, identify strategies, assume responsibility for implementation of decisions and share accountability for outcomes.	Interpersonal Processes of Care (IPC) [75]. Patient-centred decision making sub-scale (4 items). Empowerment sub-scale (5 items). Frequency of self-efficacy support from providers for self-management and healthy lifestyle on a Likert scale from "never" (1) to "almost always" (5) α = 0.91.

Respectfulness: The extent to which health professionals and support staff meet users expectations about interpersonal treatment, demonstrate respect for the dignity of patients and provide adequate privacy.	Interpersonal Processes of Care [78]. Office staff respectfulness sub-scale (4 items) on a Likert scale from "never" (1) to "almost always" (5) α = 0.93. Patient perception on quality of care - *Physical facilities - *ratings of physical facilities including cleanliness and privacy.(3 items).

**Effectiveness of Care**	

First-contact accessibility: The ease with which a person can obtain needed care (including advice and support) from the practitioner of choice within a time frame appropriate to the urgency of the problem.	Haggerty, Levesque & Roberge 2007 (unpublished) (5 items). First-contact accessibility consequences due to accessibility barriers.

Organizational accessibility or accommodation: The way primary healthcare resources are organized to accommodate a wide range of patients' abilities to contact healthcare providers and reach healthcare services. (The organization of characteristics such as telephone services, flexible appointment systems, hours of operation, and walk-in periods).	Haggerty, Levesque & Roberge 2007 (unpublished) (7 items). Measure of capacity of regular clinic to adapt to clients' ability to obtain services and differences in problem urgency. α = 0.68. PCAS Organizational access sub-scale (5 items). Rating of opening hours, ability to reach clinic by telephone, wait time for appointment on a Likert scale from "poor" (1) to "excellent" (6) α = 0.83.

Overall coordination of care between providers: The delivery of services by different providers in a timely and complementary manner such that care is connected and coherent.	Veterans Administration Outpatient Customer Satisfaction Survey [78]. Overall coordination sub-scale (6 items). Reporting of problems with information and communication linkage between all providers seen. α = 0.74.

Preventive care: Blood pressure check Pap smear Mammogram Eye examination Colorectal cancer screening Cholesterol screening Flu shot Health promotion: Providing individuals with advice and tools to make informed lifestyle decisions that improve their health and well-being.	Behavioral Risk Factor Surveillance System (BRFSS) Questionnaire - CCHS p.67 - 84 (6 items). Self reported occurrence in period of time corresponding to prevention guidelines. Good reliability and validity, except mammogram, pap smear, and cholesterol where reliability and validity is only moderate [84]. Adapted list from the Primary Care Assessment Tool (PCAT) comprehensiveness sub-scale (6 items). Recall of regular provider assessing risk for and giving advice about eating habits, alcohol consumption, smoking cessation, occupational risks, prevention of falls, emotional health, family violence on a scale of "definitely not" (1) to "definitely (4).

Chronic Illness Care: Care for chronic illness that is patient-centred, proactive, planned and includes collaborative goal setting; problem-solving and follow-up support.	Patient Assessment of Chronic Illness Care (PACIC) [80]. Sub-scales for Activation, Goal setting and Problem solving- reported frequency of provider actions and planning around self-care for chronic conditions (10 items) α = 0.82, 0.84, and 0.90 respectively (includes elements of shared-decision making).

Patient safety: Medical errors Medication review Patient education	Commonwealth fund Patient Safety questions [87]. Receiving incorrect medication or dose, or incorrect or missing test results. (2 items). Last time medication review done (1 item). IPC - frequency of being told of medication effects and side effects (2 items).

Income	Total household income from all sources. Adapted from CCHS p.280-284 (1 item).

Household Possession	Household possession of car. Owned accommodation and Registered Retirement Savings Plan (RRSP) [25] (3 items).

Social support	Help with activities of daily living, care and affection, leisure and fun activities, confiding in CCHS (4 items).

NB - Reported alpha coefficients (α) refer to statistics obtained in our validation study of different instruments (Haggerty, Levesque & Roberge 2007, unpublished).

#### Patient-centred care

Our principal measure is the Perception of Patient-Centred Care [73], adapted for usual care rather than for a single visit. We will further supplement this by exploring related dimensions such as relational continuity [74], interpersonal communication [74], shared decision-making and respectfulness [75]. All subscales refer to usual care. They are principally informative and accurate in identifying those who have a negative experience.

#### Effective care

Dimensions of effective care are patient perceptions of accessibility, coordination, prevention and health promotion, chronic illness care and patient safety, over the previous 12 months. We will measure accessibility through experienced timeliness of first contact care for urgent (but not emergency) problems [76], organizational flexibility for accommodating urgent care [77], and overall organizational accessibility [74]. Coordination is measured only in those who have seen more than one provider and measures the extent to which care is experienced as connected and coherent [78]. Measures of prevention and health promotion are measured by patient recall of the provider conducting specific clinical preventive activities and addressing the life-style habits we are measuring in our health behaviour score. The chronic illness care scale measures the extent to which elements from the Chronic Care Model [79] have been implemented by all the providers [80]. Finally, patient safety is measured by using indicators of medication errors and the receipt of risk-reduction, clinical and educational manoeuvres.

#### Multi-morbidity

will be inferred from the validated Disease Burden Morbidity Assessment [64]. Based on our conception of multi-morbidity, we propose an operational cut-off score at >10, corresponding to several diseases with minimal impact on daily living or at least two with major impact. However, a secondary objective of our analysis is to identify the threshold which is most sensitive to declining functional health, reflecting the current stage of development of multi-morbidity.

#### Poverty

will be based on the Statistics Canada low income cut-off for households, adjusted for household composition [81]. This corresponds to family incomes where the expected expenditure on food, shelter and clothing is 20 percentage points higher than for the average family. We will also generate a composite score of economic vulnerability using highest educational achievement, employment status, housing, per capita household income and perceived income adequacy.

### Analysis

The unit of analysis is the individual patient followed over the study period. We will conduct cross-sectional analysis to evaluate the comparability of our study sample with CCHS samples for Quebec and Canada. We will also confirm previously-described relationships between individual characteristics and chronic illness burden, health behaviours and functional health, as well as cross-sectional associations with healthcare.

To estimate the degree of changes in health and health functioning over time (objective 1), we will estimate annual increase in chronic illness burden, changes in health behaviours score and in functional health, which is assumed to follow a Poisson distribution.

To test our hypotheses about the effect of person-centred and effective primary healthcare on changes in functional health, health behaviours and intention to change (objective 2), we will use Poisson or ordinal logistic regression. First, we will use separate regression models to estimate the effects of patient-centred primary healthcare at T_1 _on outcomes of functional health, chronic illness burden and health behaviours at T_2 _or T_3_. We will examine the effects of individual healthcare dimensions as well as global healthcare scores to better understand the relationships with outcomes. We will examine the presence of effect modification by multi-morbidity and of poverty by testing first-order interaction terms between healthcare and multi-morbidity/poverty in the regression model.

Second, we will use structural equation modelling and path analysis (LISREL) [82] to examine the relationships between the different dependent and independent variables (objective 3). For instance, we will test the paths by which chronic illness burden and health behaviours affect functional health, finding which variables mediate these relationships. We will look for the best explanatory model by comparing the Chi-square statistic of nested models as well as goodness of fit indices, such as the Comparative Fit Index (values of 0.90 indicate good fit) and the Root Mean Square Error of Approximation (RMSEA, values lower than 0.08 indicating acceptable fit) [83].

### Sample size and statistical power

The sample size for this cohort is driven by the minimal size we need to detect a change in chronic illness burden and health behaviour change in 24 months. Estimates of incidence of chronic diseases vary by source, but in general we estimate that the annual incidence of having at least one of the physical or mental chronic illnesses of interest is approximately 100 per 1000. Assuming that incidence rates follow a Poisson distribution, a sample size of 2000 gives us 80% power to detect a rate difference of 18/1000 with α = 0.05 between any of our subgroups of interest. For path analysis, statistical power is a function of the number of variables in the model and the number of paths to be examined. Rule of thumb is that there should be 20 subjects per parameter. This sample size allows us to detect small size effects (β~0.15) in our paths of interest while controlling for individual variables.

### Ethical considerations

Participation in the research has minimal risks. Major ethical concerns are ensuring confidentiality and maintaining participation throughout the study period. Nominal information will be stored separately from data, and only the project coordinator and principal investigator will have access to the link between nominal information and the unique study identification code.

The individual's right to withdraw partially or completely will be reiterated at each new data collection effort. The consent form, which explicitly states that the study is to be carried out over several years and consists of independent consents, was approved by the scientific and ethics committees of the Centre de santé et de services sociaux de Chicoutimi, as well as the Research Ethics Committee of Hôpital Charles Lemoyne.

## Discussion

A study that follows the experience of a population sample over time will provide new and valuable information on the effectiveness of care in the population rather than in clients of selected care models. The study of how experience of primary healthcare evolves over time will be of specific value to decision-makers who implement system changes and will contribute to new knowledge in the area of measurement of healthcare experience. Focus on the patient's perspective is particularly relevant in an era of greater accountability to citizens, and reinforces the value base of primary care. Knowledge on the impact of introducing new models and on systemic effects of local configurations of healthcare and clinical governance in a population will shed new light on this issue. Repeated prospective measures provide richer information than a series of cross-sectional studies or retrospective designs. They will also generate new knowledge about the direction of relationships between care processes, patient evaluations, and individual characteristics, especially about how vulnerable persons navigate in the systems.

### Strengths and limitations

A longitudinal cohort is vulnerable to selection bias through differential loss-to-follow-up. We will collect health and socio-demographic information at recruitment to assess the extent of differential loss-to-follow-up and will conduct sensitivity analysis to examine the impact of differential losses on inferences. Some volunteer bias is also likely to occur at recruitment, however, affecting population representativeness but not the validity of analytic inferences.

Response fatigue could lead to loss-to-follow-up and information error. However, response burden needs to be weighed against the strength of a cohort design that allows us to explore various outcomes over time gaining further specificity through repeated measures.

Overall, limitations and methodological challenges are far outweighed by the unique strengths of a longitudinal cohort. It is the only design that will provide the required information on the temporal direction of effects and explore a broad set of relationships. The focus on global illness burden and all types of first-contact access is not only highly relevant to primary healthcare practice and policy, but also allows us to detect important effects despite the modest cohort size.

### Relevance and implications

To our knowledge this cohort is unique in Canada, and is also expected to yield results that are relevant internationally. Results will provide valuable information for primary healthcare clinicians on the course of chronic illness over time and the impact on health outcomes of accessible, patient-centred and effective care. A demonstration of impact will contribute to the promotion of continuous quality improvement activities at a clinical level. Finally, while considerable advances have been made in the management of specific chronic illnesses, this will make a unique contribution to effective care for persons with multiple morbidities.

## Competing interests

The authors declare that they have no competing interests.

## Authors' contributions

JF lead the design and conception of the study with MF. MF drafted the manuscript. JF, MDB, CH, CL, MP and DR participated in the critical review of the manuscript. All authors gave their final approval of the version of the manuscript submitted for publication.

## Pre-publication history

The pre-publication history for this paper can be accessed here:

http://www.biomedcentral.com/1472-6963/10/258/prepub

## References

[B1] Romanow R Building on values. The future of health care in Canada - Final report 2002 Ottawa: Commission on the Future of Health Care in Canada

[B2] Kirby MJ LeBreton M The Health of Canadians - The Federal Role: Recommendations for Reform. Volume 6, Final Report: Recommendations for Reform. 6, -392. 2002. The Standing Senate Committee on Social Affairs, Science and Technology 2002 Ottawa: Government of Canada

[B3] Government of Saskatchewan Caring for Medicare: Sustaining a Quality System. COMMISSIONER KENNETH J.FYKE, Commission on Medicare, editors. ISBN # 0-9687942-1-1, -162 2001 Regina, Saskatchewan: Policy and Planning BranchSaskatchewan Health

[B4] Primary Health Services Branch The Saskatchewan Action Plan for Primary Health Care 2002 Primary Health Services Branch

[B5] Clair M Rapport de la commission. Les solutions émergentes. Commission d'étude sur les services de santé et les services sociaux 2000 Québec: Gouvernement du Québec

[B6] Government of Ontario Looking Back,Looking Forward: The Ontario Health Services Restructuring Commission (1996-2000) A Legacy Report. The Ontario Health Services Restructuring Commission, Duncan G.Sinclair (Chair), editors 2000 Toronto, Ontario: Government of Ontario

[B7] Macinko J Starfield B Shi L Quantifying the health benefits of primary care physician supply in the United States Int J Health Serv 2007 37 111 126 10.2190/3431-G6T7-37M8-P224 17436988

[B8] Starfield B Shi L Macinko J Contribution of primary care to health systems and health Milbank Q 2005 83 457 502 10.1111/j.1468-0009.2005.00409.x 16202000 PMC2690145

[B9] Shi L Macinko J Starfield B Politzer R Xu J Primary care, race, and mortality in US states Soc Sci Med 2005 61 65 75 10.1016/j.socscimed.2004.11.056 15847962

[B10] Macinko JA Shi L Starfield B Wage inequality, the health system, and infant mortality in wealthy industrialized countries, 1970-1996 Soc Sci Med 2004 58 279 292 10.1016/S0277-9536(03)00200-4 14604614

[B11] Macinko J Starfield B Shi L The contribution of primary care systems to health outcomes within Organization for Economic Cooperation and Development (OECD) countries, 1970-1998 Health Serv Res 2003 38 831 865 10.1111/1475-6773.00149 12822915 PMC1360919

[B12] Shi L Macinko J Starfield B Wulu J Regan J Politzer R The relationship between primary care, income inequality, and mortality in US States, 1980-1995 J Am Board Fam Pract 2003 16 412 422 10.3122/jabfm.16.5.412 14645332

[B13] Shi L Macinko J Starfield B Xu J Politzer R Primary care, income inequality, and stroke mortality in the United States: a longitudinal analysis, 1985-1995 Stroke 2003 34 1958 1964 10.1161/01.STR.0000082380.80444.A9 12843344

[B14] Ettner SL The relationship between continuity of care and the health behaviors of patients: does having a usual physician make a difference? Medical Care 1999 37 547 555 10.1097/00005650-199906000-00004 10386567

[B15] Maddigan SL Majumdar SR Johnson JA Understanding the complex associations between patient-provider relationships, self-care behaviours, and health-related quality of life in type 2 diabetes: a structural equation modeling approach Qual Life Res 2005 14 1489 1500 10.1007/s11136-005-0586-z 16110929

[B16] Ettner SL The timing of preventive services for women and children: The effect of having a usual source of care Am J Public Health 1996 86 1748 1754 10.2105/AJPH.86.12.1748 9003132 PMC1380728

[B17] Gunning-Schepers LJ Hagen JH Avoidable burden of illness: How much can prevention contribute to health? Social Science & Medicine 1987 24 945 951 10.1016/0277-9536(87)90287-5 3616687

[B18] Kruse J Phillips DM Factors influencing womens' decision to undergo mammography Obstetrics & Gynecology 1987 70 744 747 3658284

[B19] McWhinney IR Primary care: core values. Core values in a changing world BMJ 1998 316 1807 1809 9624075 10.1136/bmj.316.7147.1807 PMC1113320

[B20] Howie JG Heaney D Maxwell M Quality, core values and the general practice consultation: issues of definition, measurement and delivery Fam Pract 2004 21 458 468 10.1093/fampra/cmh419 15249538

[B21] Haggerty J Burge F Lévesque JF Gass D Pineault R Beaulieu MD Santor D Operational Definitions of Attributes of Primary Health Care: Consensus Among Canadian Experts Ann Fam Med 2007 5 336 344 10.1370/afm.682 17664500 PMC1934980

[B22] Haggerty JL Pineault R Beaulieu M-D Brunelle Y Gauthier J Goulet F Rodrigue J Room for improvement: Patient experience of primary care in Quebec prior to major reforms Can Fam Physician 2007 53 1056 1057 PMC1949223 17872786

[B23] Beaulieu M-D Denis J-L D'Amour D Goudreau J Haggerty J Hudon É Jobin G Lamothe L Gilbert F Guay H Cyr G Lebeau R Implementing family medicine groups: A challenge in the reorganization of practice and interprofessional collaboration (This report can be downloaded from the Web site of the Doctor Sadok Besrour Chair in Family Medicine: wwwmedfamumontrealca/chaire_sadok_besrour/chaire/chairehtm) Doctor Sadok Besrour Chair in Family Medicine, Montreal 2006

[B24] Reinharz D Tourigny A Aubin M Bonin L Haggerty J Leduc Y Morin D St-Pierre M La réorganisation des services de premières lignes comme outil de changement des pratiques Rapport de recherche, Université Laval 2007

[B25] Pineault R Levesque J-F Tousignant P Beaulne G Hamel M Poirier L-R Raynault M-F Benigeri M Roberge D Lamarche P Haggerty J Bergeron P Dulude S Marcil M L'accessibilité et la continuité dans la population: l'influence des modèles d'organisation des services de santé de première ligne Projet financé par la Fondation canadienne de recherche sur les services de santé FCRSS RC1-1091-05 2004

[B26] Guyatt GH Feeny DH Patrick DL Measuring health-related quality of life Ann Intern Med 1993 118 622 629 8452328 10.7326/0003-4819-118-8-199304150-00009

[B27] Guyatt GH Ferrans CE Halyard MY Revicki DA Symonds TL Varricchio CG Exploration of the value of health-related quality-of-life information from clinical research and into clinical practice Mayo Clin Proc 2007 82 1229 1239 10.4065/82.10.1229 17908529

[B28] Deeg DJ Longitudinal characterization of course types of functional limitations Disabil Rehabil 2005 27 253 261 10.1080/09638280400006507 16025752

[B29] Maddigan SL Feeny DH Johnson JA Health-related quality of life deficits associated with diabetes and comorbidities in a Canadian National Population Health Survey Qual Life Res 2005 14 1311 1320 10.1007/s11136-004-6640-4 16047506

[B30] Dunlop DD Manheim LM Sohn MW Liu X Chang RW Incidence of functional limitation in older adults: the impact of gender, race, and chronic conditions Arch Phys Med Rehabil 2002 83 964 971 10.1053/apmr.2002.32817 12098157

[B31] Mo F Pogany LM Li FC Morrison HI Prevalence of diabetes and cardiovascular comorbidity in the Canadian Community Health Survey 2002-2003 Scientific World Journal 2006 6 96 105 16435038 10.1100/tsw.2006.13 PMC5944181

[B32] Canadian Institute for Health Information Improving the Health of Canadians 2004 Ottawa: Canadian Institute for Health Information

[B33] Farrell K Wicks MN Martin JC Chronic disease self-management improved with enhanced self-efficacy Clin Nurs Res 2004 13 289 308 10.1177/1054773804267878 15448281

[B34] Torrecilla M Barrueco M Jimenez RC Maderuelo J Plaza M Hernandez MM [The physician and the patient in the decision to quit smoking. Effect of the initiative on the result of the intervention] Arch Bronconeumol 2001 37 127 134 11333538 10.1016/s0300-2896(01)75034-x

[B35] Kottke TE Battista RN DeFriese GH Brekke ML Attributes of successful smoking cessation interventions in medical practice. A meta-analysis of 39 controlled trials JAMA 1988 259 2883 2889 10.1001/jama.259.19.2883 3367456

[B36] Anderson P Scott E The effect of general practitioners' advice to heavy drinking men Br J Addict 1992 87 891 900 10.1111/j.1360-0443.1992.tb01984.x 1525531

[B37] Maheswaran R Beevers M Beevers DG Effectiveness of advice to reduce alcohol consumption in hypertensive patients Hypertension 1992 19 79 84 1346121 10.1161/01.hyp.19.1.79

[B38] Wallace P Cutler S Haines A Randomised controlled trial of general practitioner intervention in patients with excessive alcohol consumption BMJ 1988 297 663 668 10.1136/bmj.297.6649.663 3052668 PMC1834369

[B39] Elley CR Dean S Kerse N Physical activity promotion in general practice--patient attitudes Aust Fam Physician 2007 36 1061 1064 18075637

[B40] Scales R Miller JH Motivational techniques for improving compliance with an exercise program: skills for primary care clinicians Curr Sports Med Rep 2003 2 166 172 12831657 10.1249/00149619-200306000-00010

[B41] Calfas KJ Long BJ Sallis JF Wooten WJ Pratt M Patrick K A controlled trial of physician counseling to promote the adoption of physical activity Prev Med 1996 25 225 233 10.1006/pmed.1996.0050 8780999

[B42] Swinburn BA Walter LG Arroll B Tilyard MW Russell DG The green prescription study: a randomized controlled trial of written exercise advice provided by general practitioners Am J Public Health 1998 88 288 291 10.2105/AJPH.88.2.288 9491025 PMC1508188

[B43] Fortin M Bravo G Hudon C Vanasse A Lapointe L Prevalence of multimorbidity among adults seen in family practice Ann Fam Med 2005 3 223 228 10.1370/afm.272 15928225 PMC1466875

[B44] Daveluy C Pica L Audet N Courtemanche R Lapointe F Enquête sociale et de santé 1998 2000 2 Québec: Institut de la statistique du Québec

[B45] Knottnerus JA Metsemakers J Hoppener P Limonard C Chronic illness in the community and the concept of 'social prevalence' Fam Pract 1992 9 15 21 10.1093/fampra/9.1.15 1634021

[B46] van den Akker M Buntinx F Metsemakers JF Roos S Knottnerus JA Multimorbidity in general practice: prevalence, incidence, and determinants of co-occurring chronic and recurrent diseases J Clin Epidemiol 1998 51 367 375 10.1016/S0895-4356(97)00306-5 9619963

[B47] Rapoport J Jacobs P Bell NR Klarenbach S Refining the measurement of the economic burden of chronic diseases in Canada Chronic Dis Can 2004 25 13 21 15298484

[B48] Fortin M Bravo G Hudon C Lapointe L Almirall J Dubois MF Vanasse A Relationship between multimorbidity and health-related quality of life of patients in primary care Qual Life Res 2006 15 83 91 10.1007/s11136-005-8661-z 16411033

[B49] Fortin M Bravo G Hudon C Lapointe L Dubois MF Almirall J Relationship between psychological distress and multimorbidity of patients in family practice Ann Fam Med 2006 4 417 422 10.1370/afm.528 17003141 PMC1578652

[B50] Fortin M Hudon C Bayliss EA Soubhi H Lapointe L Caring for body and soul: The importance of recognizing and managing psychological distress in persons with multimorbidity Int'l J Psychiatry in Medicine 2007 37 1 9 10.2190/41X8-42QW-2571-H20G 17645193

[B51] Fortin M Dubois M-F Hudon C Soubhi H Almirall J Multimorbidity and quality of life: a closer look Health Qual Life Outcomes 2007 5 52 10.1186/1477-7525-5-52 17683600 PMC2042974

[B52] Fortin M Hudon C Dubois M-F Almirall J Lapointe L Soubhi H Comparative assessment of three different indices of multimorbidity for studies on health-related quality of life Health Qual Life Outcomes 2005 3 74 10.1186/1477-7525-3-74 16305743 PMC1310518

[B53] Hagenaars A de Vos K The Definition and Measurement of Poverty The Journal of Human Resources 1988 23 211 221 10.2307/145776

[B54] Wagle U Rethinking poverty: definition and measurement 2002 Unesco: Blackwell Publishers

[B55] Bryant H Robson PJ Ullman R Friedenreich C Dawe U Population-based cohort development in Alberta, Canada: a feasibility study Chronic Dis Can 2006 27 51 59 16867239

[B56] Haggerty J Burge F Beaulieu M-D Gass D Lévesque J-F Pineault R Santor D Evaluating the quality of primary care from the consumer perspective: development of instruments adapted to the Canadian context Projet financé par les Instituts de recherche en santé du Canada 2004

[B57] Canadian Internet Use Survey http://www.statcan.gc.ca/daily-quotidien/060815/dq060815b-eng.htm

[B58] Dillman DA Mail and Telephone Surveys: The Total Design Method 1978 New York: John Wiley and Sons

[B59] Dillman DA Mail and Internet Surveys. The tailored design method 2000 2 New York: John Wiley & Sons, Inc

[B60] Ware JE Kosinski M T-BDMGB How to Score Version 2 of he SF-12 Health Survey (With a Supplement Documenting Version 1) 2002 Lincoln, RI: Quality Metric Incorporated

[B61] The International Quality Of Life Assessment Project http://www.iqola.org/project.aspx#top

[B62] Kopec JA Willison KD A comparative review of four preference-weighted measures of health-related quality of life J Clin Epidemiol 2003 56 317 325 10.1016/S0895-4356(02)00609-1 12767408

[B63] Kopec JA Schultz SE Goel V Ivan WJ Can the health utilities index measure change? Med Care 2001 39 562 574 10.1097/00005650-200106000-00005 11404641

[B64] Bayliss EA Ellis JL Steiner JF Subjective assessments of comorbidity correlate with quality of life health outcomes: Initial validation of a comorbidity assessment instrument Health and Quality of life Outcomes 2005 3 51 10.1186/1477-7525-3-51 16137329 PMC1208932

[B65] Centers for Disease Control and Prevention (CDC) Behavioral Risk Factor Surveillance System Survey Questionnaire 2007 Atlanta, Georgia: U.S. Department of Health and Human Services, Centers for Disease Control and Prevention

[B66] Enquête de santé du Saguenay-Lac-Saint-Jean 2007, Rapport sommaire http://www.santesaglac.gouv.qc.ca/publication6.html

[B67] Laforge RG Velicer WF Richmond RL Owen N Stage distributions for five health behaviors in the United States and Australia Prev Med 1999 28 61 74 10.1006/pmed.1998.0384 9973589

[B68] Laforge RG Rossi JS Prochaska JO Velicer WF Levesque DA McHorney CA Stage of regular exercise and health-related quality of life Prev Med 1999 28 349 360 10.1006/pmed.1998.0429 10090864

[B69] Prochaska JO Norcross JC Stages of Change Psychotherapy & Psychosomatics 2001 38 443 448

[B70] Plotnikoff RC Bercovitz K Rhodes RE Loucaides CA Karunamuni N Testing a conceptual model related to weight perceptions, physical activity and smoking in adolescents Health Educ Res 2007 22 192 202 10.1093/her/cyl065 16861363

[B71] Rhodes RE Plotnikoff RC Can current physical activity act as a reasonable proxy measure of future physical activity? Evaluating cross-sectional and passive prospective designs with the use of social cognition models Prev Med 2005 40 547 555 10.1016/j.ypmed.2004.07.016 15749137

[B72] Sarkin JA Johnson SS Prochaska JO Prochaska JM Applying the transtheoretical model to regular moderate exercise in an overweight population: validation of a stages of change measure Prev Med 2001 33 462 469 10.1006/pmed.2001.0916 11676588

[B73] Stewart M Belle Brown J Donner A McWhinney IR Oates J Weston WW Jordan J The Impact of Patient-Centered Care on Outcomes The Journal of Family Practice 2000 49 796 804 11032203

[B74] Safran DG Kosinski M Tarlov AR Rogers WH Taira DH Lieberman N Ware JE The Primary Care Assessment Survey: tests of data quality and measurement performance Med Care 1998 36 728 739 10.1097/00005650-199805000-00012 9596063

[B75] Stewart AL Nápoles-Springer A Pérez-Stable EJ Interpersonal processes of care in diverse populations Milbank Q 1999 77 305 339 10.1111/1468-0009.00138 10526547 PMC2751132

[B76] Shi L Starfield B Xu J Validating the Adult Primary Care Assessment Tool Journal of Family Practice 2001 50 161

[B77] Gauthier J Haggerty J Pineault R Lamarche P Morin D Sylvain H Lévesque J-F Modèles d'organisation des services de santé primaire et accès aux services requis par les communautés rurales, éloignées et isolées du Québec Projet subventionné par le FCRSS (Fondation canadienne de la recherche sur les services de santé) 2003

[B78] Borowsky SJ Nelson DB Fortney JC Hedeen AN Bradley JL Chapko MK VA community-based outpatient clinics: performance measures based on patient perceptions of care Med Care 2002 40 578 586 10.1097/00005650-200207000-00004 12142773

[B79] Wagner EH Austin BT Von Korff M Organizing care for patients with chronic illness Milbank Q 1996 74 511 544 10.2307/3350391 8941260

[B80] Glasgow RE Wagner EH Schaefer J Mahoney LD Reid RJ Greene SM Development and validation of the Patient Assessment of Chronic Illness Care (PACIC) Med Care 2005 43 436 444 10.1097/01.mlr.0000160375.47920.8c 15838407

[B81] Income Statistics Division Low Income Cut-offs for 2005 and Low Income Measures for 2004 Ottawa: Statistics Canada 2004

[B82] Jöreskog KG Sörbom D LISREL 8: Users' reference guide 1993 Chicago: Scientific Software International, Inc

[B83] Browne MW Cudeck R Bollen KA, Long JS Alternative ways of assessing model Testing Structural Equations models 1993 Newbury Park, Calif: Sage 136 162

[B84] Nelson DE Holtzman D Bolen J Stanwyck CA Mack KA Reliability and validity of measures from the Behavioral Risk Factor Surveillance System (BRFSS) Soz Praventivmed 2001 46 Suppl 1 S3 S42 11851091

[B85] Kessler RC Andrews G Colpe LJ Hiripi E Mroczek DK Normand SL Short screening scales to monitor population prevalences and trends in non-specific psychological distress Psychol Med 2002 32 959 976 10.1017/S0033291702006074 12214795

[B86] Kessler RC Barker PR Colpe LJ Epstein JF Gfroerer JC Hiripi E Screening for serious mental illness in the general population Arch Gen Psychiatry 2003 60 184 189 10.1001/archpsyc.60.2.184 12578436

[B87] Davis K Schoen C Schoenbaum SC Holmgren AJ Kriss JL Mirror, Mirror on the Wall: An Update on the Quality of American Health Care Through the Patient's Lens 2006 The Commonwealth Fund

